# Construction of Large-Volume Tissue Mimics with 3D Functional Vascular Networks

**DOI:** 10.1371/journal.pone.0156529

**Published:** 2016-05-26

**Authors:** Tae-Yun Kang, Jung Min Hong, Jin Woo Jung, Hyun-Wook Kang, Dong-Woo Cho

**Affiliations:** 1 Department of Biomedical Engineering, Yale University, New Haven, Connecticut, United States of America; 2 Department of Mechanical Engineering, Pohang University of Science and Technology (POSTECH), Pohang, Gyungbuk, Korea; 3 Department of Biomedical Engineering, School of Life Sciences, Ulsan National Institute of Science and Technology (UNIST), Ulsan, Korea; University of Connecticut Health Center, UNITED STATES

## Abstract

We used indirect stereolithography (SL) to form inner-layered fluidic networks in a porous scaffold by introducing a hydrogel barrier on the luminal surface, then seeded the networks separately with human umbilical vein endothelial cells and human lung fibroblasts to form a tissue mimic containing vascular networks. The artificial vascular networks provided channels for oxygen transport, thus reducing the hypoxic volume and preventing cell death. The endothelium of the vascular networks significantly retarded the occlusion of channels during whole-blood circulation. The tissue mimics have the potential to be used as an *in vitro* platform to examine the physiologic and pathologic phenomena through vascular architecture.

## Introduction

Tissue engineering has led to *in vitro* construction of tissues/ organs and may ameliorate the limited supply of organs for transplantation. Currently only a few types of organs such as skin[1,2], bladders[3,4], and tracheas[5,6] have been engineered for clinical application. Unlike living tissues *in vivo*, cell viability and functions cannot be sustained in the core of dense engineered tissue *in vitro* [7–9] because diffusion alone supplies nutrients and oxygen to cells within engineered tissue; the lack of adequate mass transport leads to necrotic cell death in the core. The limitation of diffusion becomes increasingly critical as the volume and cell population of engineered tissue increase. Thus bioengineers are seeking ways to incorporate microcirculation into engineered tissues [10–16]; methods include induction of angiogenesis by biomolecular cues, and formation of microvasculature. The introduction of microfabrication technologies to tissue engineering has allowed construction of perfusable channels in engineered tissues [13,17–19]; this approach has the potential to achieve stable mass transport from the initiation of the cell culture.

To construct functional channels for this purpose, emphasis should be placed on developing endothelized channels in an engineered tissue for metabolite transport *in vivo*. Soft lithography, micromachining, and micromolding technologies have been utilized to fabricate two-dimensional (2D) endothelized channels with biomaterials [13,17,18]. 3D printing of carbohydrate fibers and rapid casting have been used to fabricate endothelized lattice channels in 3D hydrogel in the present of living cells [19]. To realize the complex branching patterns of vascular networks, 3D printing technology based on layer-by layer deposition has enabled the construction of 3D vascular networks with various branching patterns and angles[20,21]. However, cells should be repopulated after completing the structure fabrication because the process entails use of cytotoxic organic solvents. Thus, challenges remain in controlling the position of repopulated cells to form tissues with spatially distinct endothelium on the lumen of the porous monolithic structure fabricated by the technology.

The objective of this study was to use stereolithography (SL) based on photopolymerization [22] to construct 3D functional vascular networks in tissue mimics, and to establish a basis for large-volume tissue regeneration. Although SL technology requires use of photocurable materials, a wide range of biomaterials can be used with indirect method [23]. The work led to a well-designed molding process and successful fabrication of scaffolds using PLGA, PLLA, PCL, chitosan, alginate, and bone cement with the resolution of 50 ~70 um. Here, we modified indirect SL technology to enable formation of an inner-layered fluidic network in a porous scaffold by introducing a hydrogel barrier on the luminal surface. The scaffold containing inner-layered fluidic networks was turned into a tissue mimic containing vascular networks by seeding it separately with human umbilical vein endothelial cells (HUVECs) and human lung fibroblasts (HLFs). The effects of artificial vascular networks on oxygen delivery and cell viability were assessed under perfusion culture, and the early performance of vascular networks beyond 24 h in a physiological environment was investigated using whole-blood perfusion as a surrogate for transplantation.

## Materials and Methods

### Design of a microfluidic network system for a porous scaffold

We designed 3D fluidic network models for a cylindrical scaffold (10 mm in diameter × 10 mm in length) based on an algorithm introduced previously [8]. At bifurcation lesions, the parent branch and its two daughter branches lay in the same plane and the branch opening half-angles did not exceed $\frac{\pi }{4}$, which is the maximum value in the physiologically relevant range. The inlet diameter was set to be 2 mm and the daughter diameters were determined using Murray’s law[24]. The same numerical analysis was performed to evaluate oxygen transport by 3D fluidic networks in the 3D large-volume scaffold.

### Fabrication of Scaffolds with Inner-layered Fluidic Network

**Fig 1:**
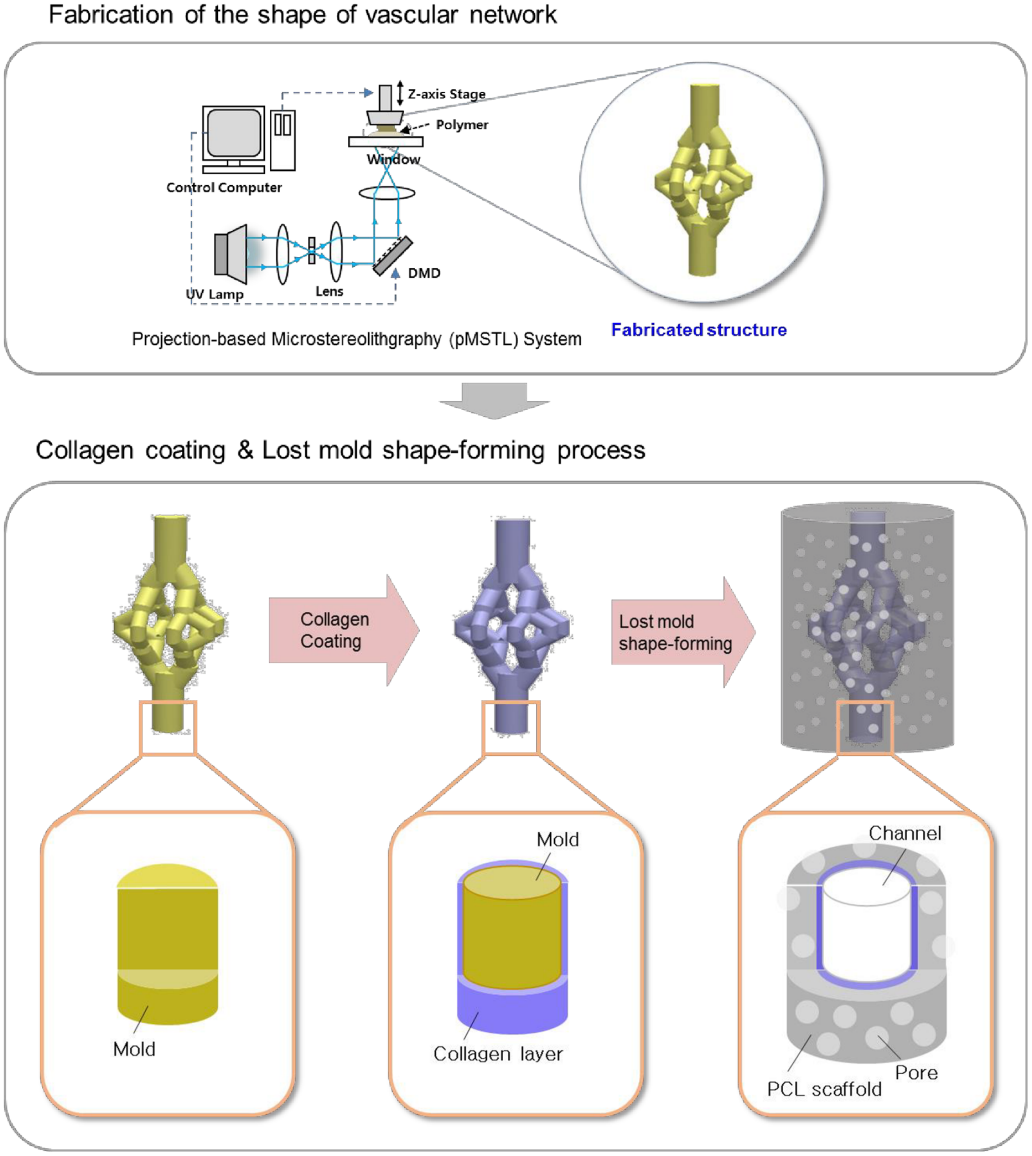
Modified indirect-MSTL technology for the porous scaffold containing a fluidic network system with collagen inner layer. The magnified schematics in the collagen coating and lost mold shape-forming process show the internal structure in each process.

Indirect-SL fabrication [8,23] was proposed to construct dual-pore scaffolds having designed pores and local pores together by combining SL technology and sacrificial molding process. A sacrificial mold having an inverse shape of global pores is fabricated from an alkali-soluble photopolymer using the SL technology. Then the mold is filled with biomaterials and local pores are formed by traditional methods such as phase inversion and salt leaching techniques. Finally, the sacrificial mold is removed and designed global pores and irregular local pores are formed within a structure. In this study, the indirect-SL fabrication technique was modified to construct fluidic networks having a collagen inner layer in a porous scaffold (Fig 1). As described in detail previously, first, projection image data were created from computer-aided design models of the designed fluidic networks [25]. Second, the shape of the fluidic networks was fabricated from an alkali-soluble photopolymer (44 wt% N,N-dimethyl-acrylamide, 44 wt% methacrylic acid, 12 wt% poly(vinyl pyrrolidone))[26] using a projection-based microstereolithography (pMSTL) system. Third, the fabricated structure was dipped in a collagen solution (2 v/v%, Koken atelocollagen implant, Koken, Japan) and the collagen was cross-linked in N-hydroxysuccinimide(NHS)/N-(3-dimethylaminopropyl)-N’-ethylcarbodiimide (EDC) solution in ethanol (NHS/EDC at 1:1 each 10 mg/ml in 95% ethanol). Fourth, polycaprolactone (PCL; Polysciences, Inc., Warrington, PA, USA; Mw 43,000–50,000, 20% w/v) solution in chloroform was mixed with sodium chloride particles which were sieved through a 300-μm mesh, and the mixture was poured into the mold and placed in pure isopropyl alcohol to remove the chloroform. Then the sacrificial mold and salt were dissolved using 0.5 N NaOH for 8 hours. The dissolution time and the effect of residues of sacrificial mold were investigated in our previous work[23] and this protocol was established based on the results. Because the PCL surface is hydrophobic and lacks active sites to immobilize collagen molecules, the surface of the collagen layer and porous scaffold are in contact without any interfacial anchoring. Hydrolysis of PCL by NaOH during the step of removing the sacrificial mold can break the ester bonds in PCL; this process introduces active sites on the surface of PCL by producing carboxylic groups and hydroxyl groups [27,28]. After removing the mold in NaOH solution, the structure was immersed in EDC/NHS solution again to induce amide bonds between the PCL scaffold and the collagen layer. The final PCL structure was carefully washed with distilled water three times for total 24 h. To observe the surface, scaffolds were sputter-coated with gold and images were taken with scanning electron microscopy (SEM; Hitachi SU-6600; Hitachi, Tokyo, Japan). To observe the inner architecture, scaffolds were cross-sectioned with a cryostat (Leica, Wetzlar, Germany) and images of bifurcation lesions were taken using micro-CT.

### Assessment of Collagen Denature by Fourier transform infrared (FT-IR) spectroscopy

In the fabrication process (Fig 1), collagen is exposed in several solvents such as isopropyl alcohol, NaOH, and chloroform. To determine whether the collagen was denatured by the solvents, infrared spectra were obtained for the raw and processed collagen. The raw collagen film was prepared after crosslinking in EDC/NHS solution. The processed collagen film was prepared by immersing the raw collagen in isopropyl alcohol for 2 d, NaOH solution for 8 h, and chloroform for 2 d one after another. Spectra were obtained using a Fourier transform infrared spectrometer (M1200, MIDAC Co. Ltd).

### Construction of 3D Artificial Tissue Mimics

**Fig 2:**
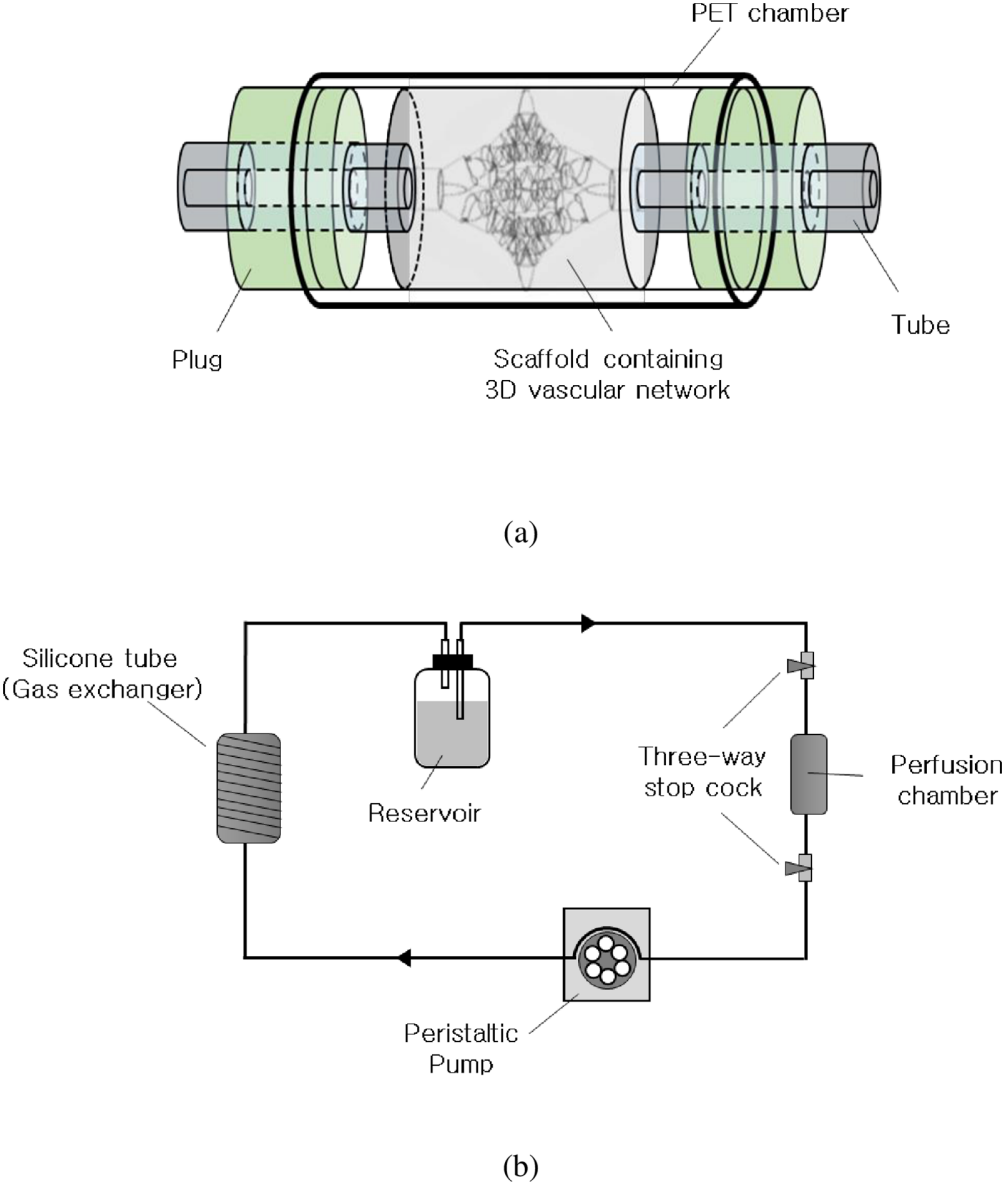
Cell culture system. (a) A perfusion chamber for loading a scaffold, and (b) perfusion culture system.

The porous scaffold containing inner-layered fluidic networks was turned into a tissue mimic containing vascular networks by seeding it with HUVECs and HLFs cells in two steps. HUVECs and HLFs were purchased from ATCC and labeled with DiI (red) and DiO (green) dyes, respectively, in accordance with the manufacturer’s (Molecular probes, Invitrogen) instructions. Briefly, cells were dissociated by treatment with trypsin, then resuspended in 1 ml medium. The cell suspensions were mixed with 5 μl of DiI or DiO cell labeling solutions, then incubated at 37°C for 30 min. First, a suspension of HUVECs was injected into the inlet of the fluidic network until the suspension filled the whole network. After shaking and incubating for 3 h at 37°C and 5% CO_2_, HUVECs were attached to the luminal surface of the fluidic network. Second, HLFs were seeded onto the scaffolds surrounding the fluidic network by using a vacuum-aided seeding technique [29] to homogeneously distribute the cells across the surface and thickness of the scaffold. Then the scaffolds were loaded in perfusion chambers (Fig 2a) and cultured for 6 days in the perfusion system (Fig 2b). The perfusion system composed of a peristaltic pump, medium reservoir, gas permeable silicon tube as a gas exchanger, and chamber. The PET chamber was designed to have the same diameter with scaffolds and plugged with silicon rubbers which were connected to tubes for perfusion.

The pimonidazole conjugation assay was used to detect hypoxic cells in the tissue mimics. Pimonidazole is reductively activated in hypoxic cells and forms stable covalent adducts with thiol (sulphydryl) groups in proteins, peptides and amino acids. According to the manufacturer’s protocol, the cells having those adduct are detected by immunochemical means. First, a pimonidazole was added to the cell culture media at day 3 and incubated for 36 h. The tissue mimics were fixed for 30 min in 10% formalin, frozen, and embedded in O.C.T. compound embedding medium (Tissue-Tek, Sakura Fineteck Inc, Torrance, CA, USA). Then, 4-μm sections were prepared from the frozen block with a cryostat. In the process, the collagen layer was somewhat detached from the scaffold surface but maintained the shape of fluidic network. Samples were mounted with DAPI and images were taken under a fluorescence microscope (LX71; Olympus, Tokyo, Japan).

Cell apoptosis was detected using an assay for degradation of DNA (DeadEnd Fluorometric TUNEL System, Promega). Cell-seeded scaffolds were washed and fixed in 4% paraformaldehyde according to the manufacturer’s protocols. After preparing 4-μm sections as described above, fixed cells were permeabilized with Triton X-100, then labeled with the Terminal Deoxynucleotidyl Transferase (TdT) enzyme. Apoptotic cells were identified by green fluorescence using a fluorescence microscope. Images were acquired from three different positions for the analysis.

### Statistical Analyses

Statistical analyses were performed via one-way analysis of variance with a post hoc Tukey test using MINITAB version 17 (State College, PA, USA). Differences between groups were considered statistically significant at P < 0.05.

### Whole Blood Circulation as a Surrogate Model of Transplantation

To investigate the patency of artificial vascular networks in physiological environment, we adopted whole-blood perfusion as a surrogate for transplantation [30]. According to the IRB regulations established by Korean Ministry of Health and Welfare, this research involving the use of anonymous human tissue specimens is exempt from the requirement for IRB approval. The whole-blood perfusion was performed in the perfusion culture system (Fig 2), using whole blood as a perfusate instead of culture medium. Fresh blood was collected in vacutainer tubes containing sodium citrate (BD, USA) from a healthy adult volunteer free of aspirin or other drugs that could bias the results. The blood was taken specifically for this study. After 6 d of culture, the culture medium was washed away with phosphate buffer saline and whole blood was circulated for 24 h, during which it was replenished three times. The patency of artificial vascular networks was observed by the alteration of flow rate. The tissue mimics were fixed after 24 h of whole-blood circulation and 10-μm sections were prepared for macroscopic images and hematoxylin and eosin (H&E) staining.

## Results

### Optimal Design of the Fluidic Network System based on the Oxygen Transport Simulation

**Fig 3:**
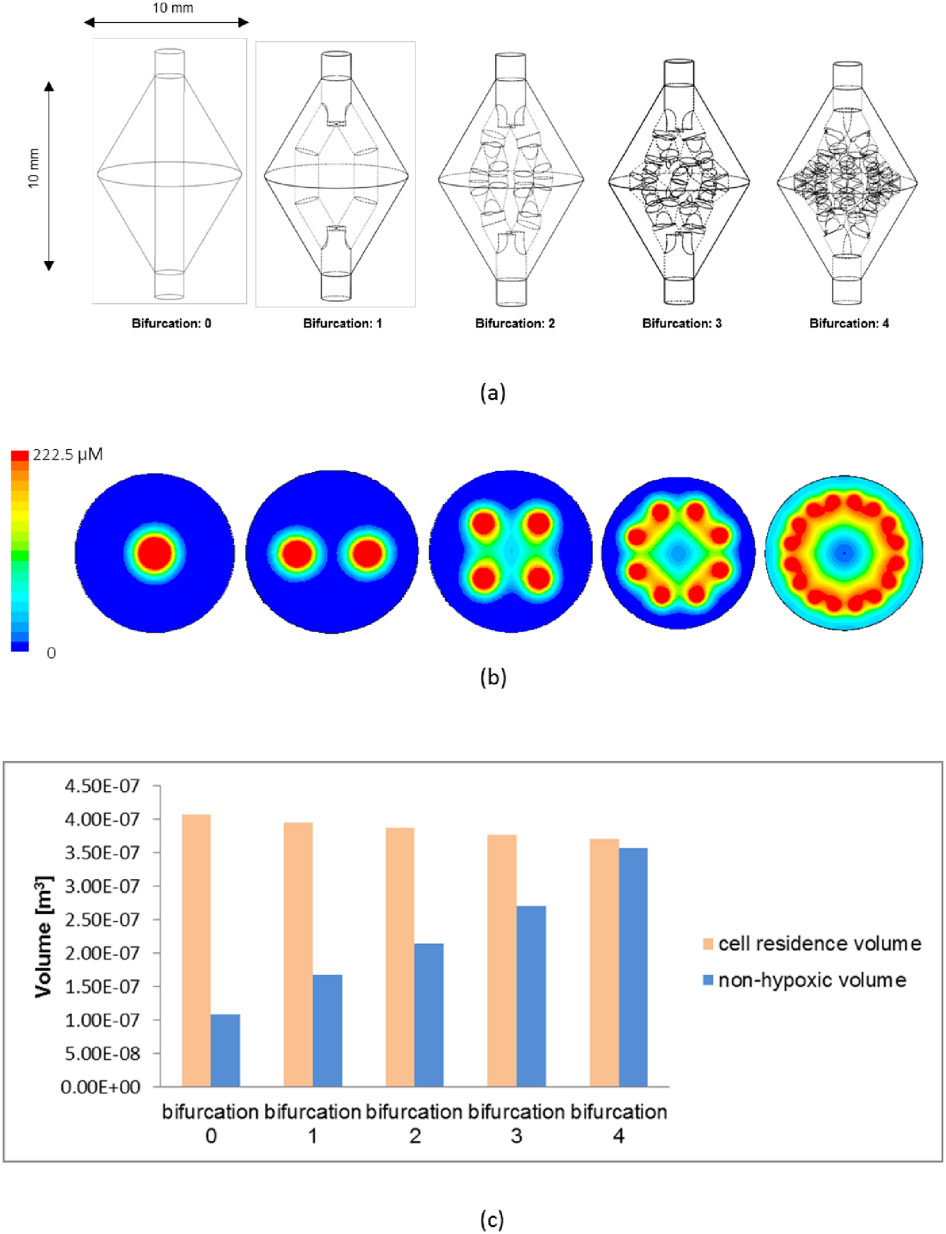
Evaluation of the 3D fluidic network based on an oxygen transport simulation. (a) Designs of 3D fluidic network, (b) simulated distribution of oxygen concentration in Bifurcation:4, and (c) simulated changes in cell resident volume and non-hypoxic volume according to the number of bifurcations.

As the number of bifurcations in the 3D fluidic network increased, the oxygen distribution (Fig 3a and 3b) became increasingly uniform, and calculated resident cell volume decreased and non-hypoxic volume increased (Fig 3c); 96.5% of the cell resident volume became non-hypoxic at “Bifurcation: 4”. Thus, further bifurcation was judged to be unnecessary and “Bifurcation: 4” was selected as the model for scaffold fabrication.

### Scaffolds with Inner-layered Fluidic Network

**Fig 4:**
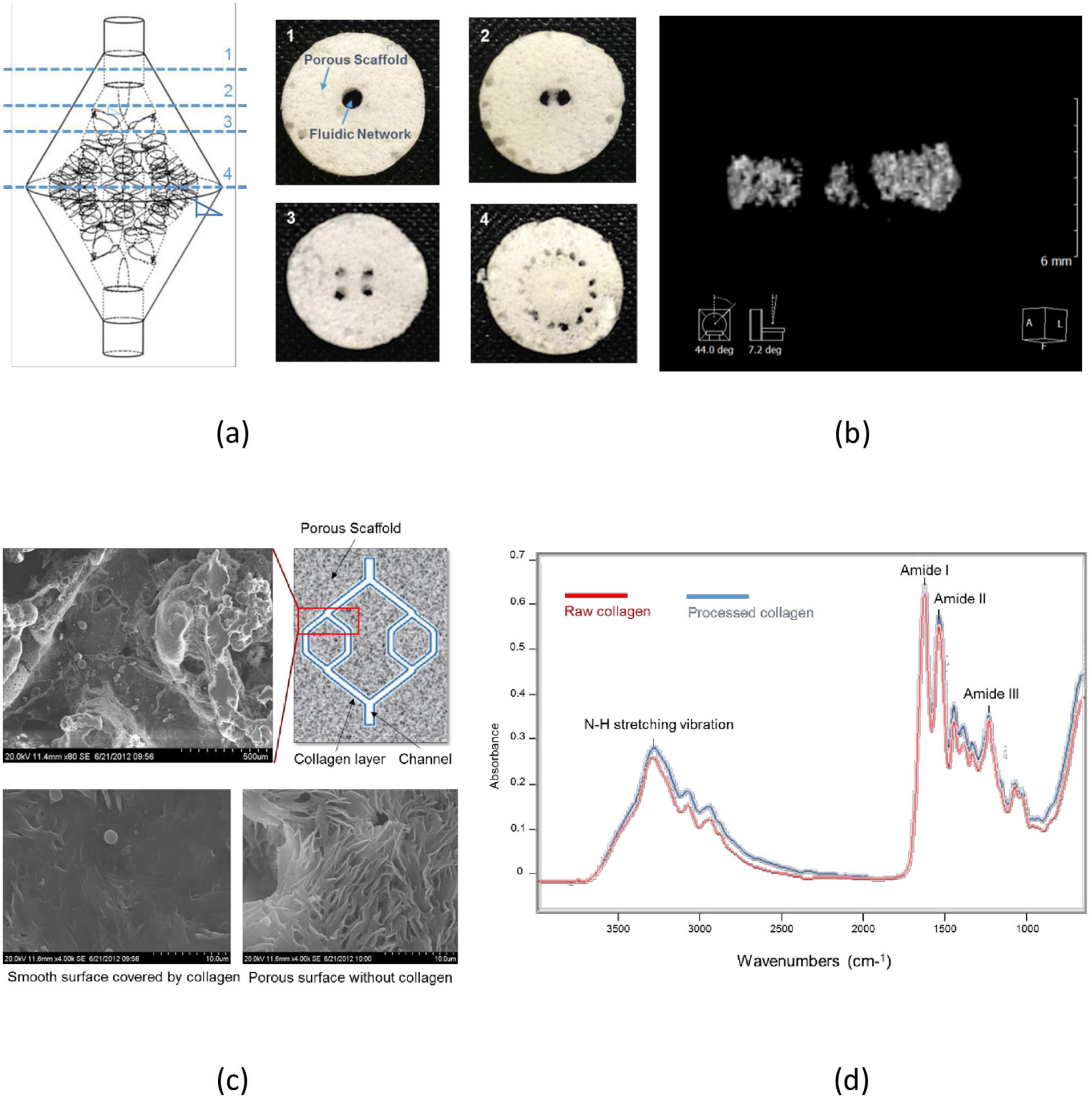
Porous scaffold containing inner-layered 3D fluidic network. (a) Cross sections acquired at the dashed lines, (b) bifurcating channels at the 1^st^ bifurcation, (c) fluidic network with collagen inner layer, (d) FT-IR spectrums of raw and processed collagen films.

Porous scaffolds with 3D fluidic network were successfully fabricated using the combination of indirect-SL technology and salt leaching. Because the PCL and salt were mixed in the same ratio as in the previous study [8,31], we assumed that the porosity and hydraulic permeability of the scaffold except the fluidic network part are 67 ± 2.9% and 1.51 ± 0.074 × 10^−12^ m^2^, respectively. Pores were formed in the spaces occupied by sodium chloride crystals. Although the sodium particles were sieved through a 300-μm mesh, the pores sizes are irregular as shown in Fig 4c. This is the inherent limitation of salt leaching method. Channels were successfully fabricated at each bifurcation (Fig 4a) and the measured diameters were 150 μm ~ 210 μm less than the designed diameters (Table 1) due to shrinkage of the 3D fluidic network mold during collagen coating. The micro-CT image shows the bifurcating channels in the porous scaffold (Fig 4b). Because the plane of bifurcation varies with position, the representative image was taken from the “Bifurcation:4” model. The channel lumens were covered with thin collagen layers and the pores were unexposed on the lumen (Fig 4c). FT-IR spectra (Fig 4d) of films show the N-H stretching vibration (3330–3310 cm^-1^) that is the major characteristic peak of collagen, and also an amide I band (1640–1660 cm^-1^), an amide II (1535–1550 cm^-1^), and an amide III (1230–1270 cm^-1^) band. Compared with the spectra of raw collagen, the peaks of processed collagen are present at similar positions; therefore the solvents did not significantly denature it during the fabrication process.

**Table 1:** Designed and Fabricated Channel Diameters.

	0 bifurcation	1^st^ bifurcation	2^nd^ bifurcation	3^rd^ bifurcation	4^th^ bifurcation
Designed diameter [mm]	2	1.59	1.26	1	0.79
Fabricated diameter [mm]	1.85	1.38	1.02	0.79	0.57

### Engineered-Tissue Containing Vascular Networks

To develop artificial vascular networks from the fluidic networks, endothelization on the luminal surface was attempted. The collagen layer was assumed to have two functions on the luminal surface. First, the collagen layer can improve endothelization [32]. PCL, which is the material of the porous scaffold, is hydrophobic and lacks active sites for biomolecule immobilization and cell attachment. A collagen layer on the PCL surface can improve the initial cell attachment and promote formation of a confluent endothelial layer. Second, the collagen layer can prevent endothelial cell migration from the luminal surface into the porous scaffold. The pore size of the scaffold is ~300 μm, whereas the size of endothelial cell is ~10 μm; therefore, cells could infiltrate and migrate through pores on the luminal surface when cell suspension is injected into the fluidic network for seeding. The collagen layer can be a barrier between endothelial cells and porous scaffold.

**Fig 5:**
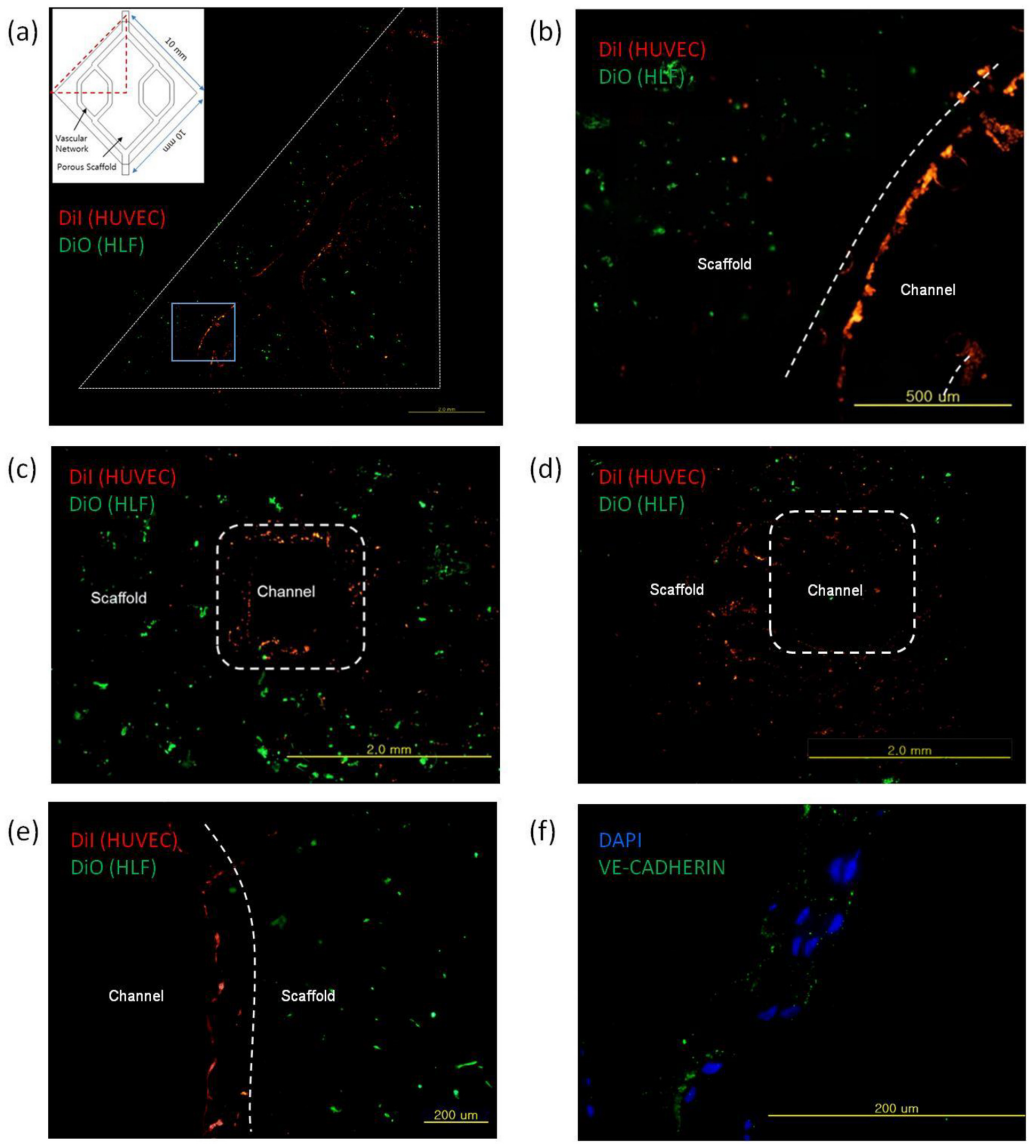
Tissue mimics containing vascular networks. (a) Distributions of HUVEC and HLF separated by collagen layer in the 2D vascular network model, (b) magnified image of (a) at the position indicated by blue square, (c) HUVEC and HLF separated by collagen layer, (d) HUVEC and HLF mixed near lumen without collagen layer, (e) HUVEC and HLF separated by collagen layer in the 3D vascular network model, (f) endothelial cell lining with adherens junction.

After 6 d of perfusion culture most endothelial cells were on the luminal surface, whereas fibroblasts were distributed in the porous scaffold region (Fig 5a–5c). Some endothelial cells were present in the porous scaffold region (Fig 5d), possibly due to overflow of cell suspension during the injecting process or leakage through holes of the collagen layer. Stained image (Fig 5e) from the cross section of the 3D vascular network shows a similar cell distribution on the luminal surface. Because of the opacity of PCL, the formation of adherence junctions between endothelial cells in the form of 3D border lines could not be seen easily, but the VE-Cadherin staining from the cross section of 3D vascular network shows evidence of an endothelial cell lining with intracellular junctions (Fig 5f), which defines the endothelial barrier functions [33,34].

### Functions of Vascular Networks in Tissue Mimics

**Fig 6:**
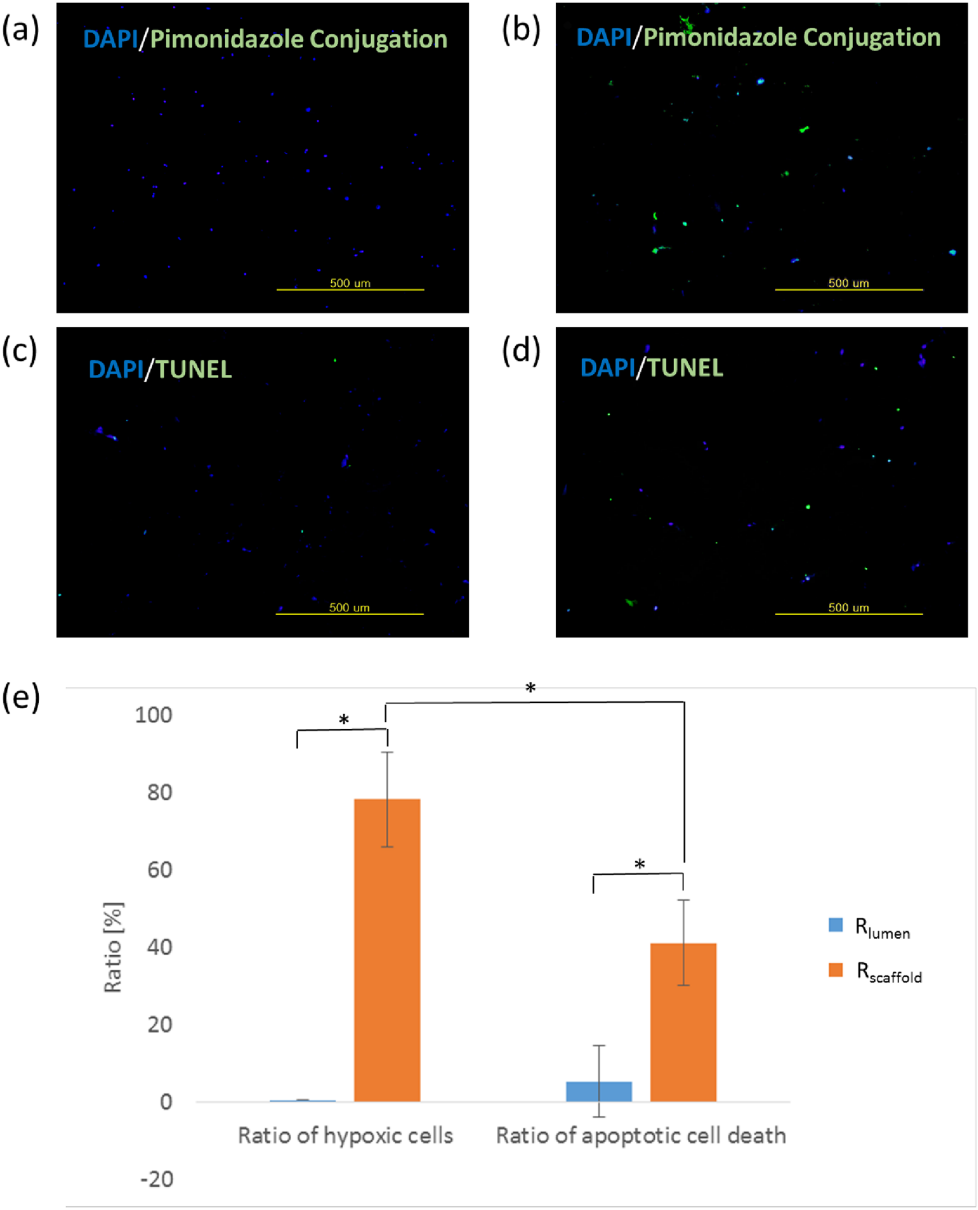
Correlation of ratios of hypoxic cells and apoptotic cell death. Ratios of hypoxic cells in (a) near lumen (*R*_*lumen*_) and (b) in the scaffold region (*R*_*scaffold*_), ratios of apoptotic cell death (c) near lumen (*R*_*lumen*_) and (d) in the scaffold region (*R*_*scaffold*_), (e) quantified result from (a)-(d), *P<0.05.

To directly investigate whether hypoxia induced cell death, we compared the percentages of apoptotic cell death and hypoxic cells in two separate areas, *R*_*lumen*_ and *R*_*scaffold*_ in the cross sections at the 1^st^ bifurcation. *R*_*lumen*_ and *R*_*scaffold*_ were defined as the area within and outside 1 mm from the channel lumen, respectively. The percentage of apoptotic cell death significantly increased to 41.2% in *R*_*scaffold*_ where the ratio of hypoxic cell was 78.2% (Fig 6).

**Fig 7:**
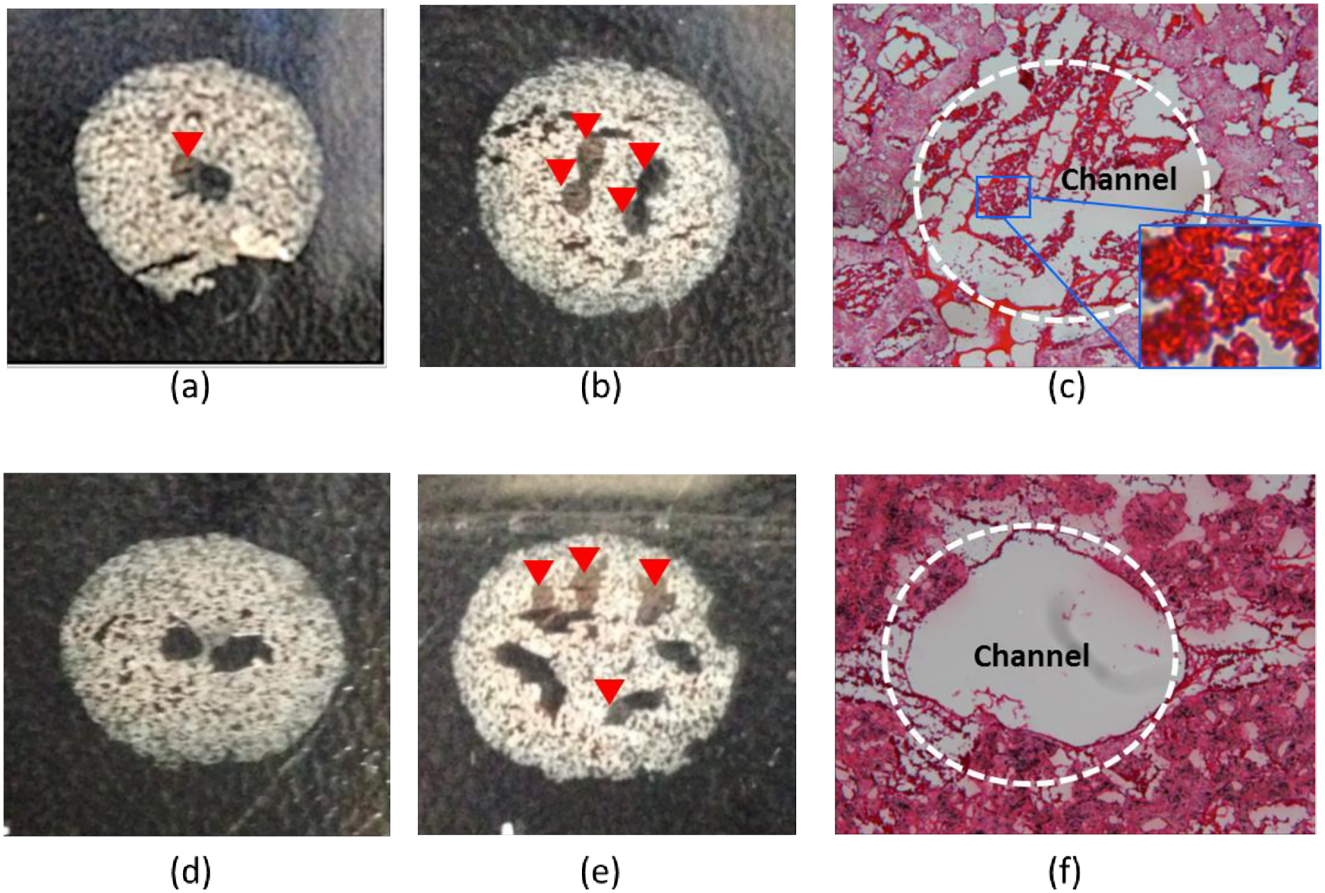
Patency of artificial vascular networks after whole-blood circulation. Occluded channels of *VN*_*non-collagen layer*_ at the1^st^ bifurcation (a) and the 2^nd^ bifurcation (b), perfectly open (d) and partially open (e) channels of *VN*_*collagen layer*_ at the1^st^ bifurcation and the 3^rd^ bifurcation, respectively (The red arrows indicate the occluded channels), H&E image of *VN*_*non-collagen layer*_ showing the thrombus consisting of red blood cells, (c) and H&E image of *VN*_*collagen layer*_ showing patent channel (f) at the 3^rd^ bifurcation.

During whole-blood perfusion, the chamber containing tissue mimics without collagen layer (*VN*_*non-collagen layer*_) filled with bubbles soon after perfusion and the flow rate became extremely slow, but the chamber containing tissue mimics with collagen layer (*VN*_*collagen layer*_) started to fill with bubbles after 24 h (images are not shown). Macroscopic pictures of cross sections of *VN*_*non-collagen layer*_ and *VN*_*collagen layer*_ demonstrate that the channels of *VN*_*non-collagen layer*_ started to be occluded at the 1^st^ bifurcation and were almost occluded at the 2^nd^ bifurcation (Fig 7a and 7b). In contrast, the channels of *VN*_*collagen layer*_ were perfectly patent at the 1^st^ bifurcation and half of channels were patent even at the 3^rd^ bifurcation (Fig 7d and 7e). H&E staining images definitely reveal that the occlusion was caused by red blood cells, which are the primary factors of thrombosis (Fig 7c and 7f). The formation of bubbles during perfusion was assumed to be induced by blood flowing thorough the micropores in the scaffolds instead of through the occluded channels.

## Discussion

We have performed a series of studies to develop tissue mimics containing functional vascular networks which are designed based on oxygen transport simulation [8,31]. First, the effective diffusion coefficient in a cell-seeded scaffold was experimentally measured [31]. Then a procedure to design an effective fluidic network system for an engineered tissue was proposed based on the coefficient[8]. And the reliability of the procedure was demonstrated by experiments using scaffolds containing the 2D microfluidic network system. Herein, we eventually developed a large-volume scaffold containing 3D functional vascular network that allows stable mass transport within the scaffold. This study not only adapts our strategy to design effective fluidic network based on numerical analysis in 3D, but also shows the technical development to construct tissue mimics containing endothelium as artificial vascular networks. Contrary to other reports[13,19], cells were repopulated after completing the fabrication process due to the use of NaOH solvent for removing the sacrificial mold. However, the results demonstrated that cells could be evenly repopulated in the large-volume scaffold with vacuum-aided seeding[31], and that spatially-distinct endothelium could be formed on the lumen of the porous monolithic structure. Moreover, the technology can fabricate 3D channels with various branching patterns and angles, can use a wide range of biomaterials, from synthetic polymers to natural polymers, and can be combined with other technologies such as gas foaming and phase inversion to form micropores in the scaffold.

In our previous study[8], the distance from the surface exposed to medium to the point at which pimonidazole staining first occurred was 1.2mm. This value was set to be the limit of non-hypoxic area and the volume of a cylindrical scaffold (10 mm in diameter × 10 mm in length) was determined to exceed this limit as well as to be close to the maximum volume which can be fabricated with our current system. Highly branched fluidic networks can densely pervade a region, but the increase in channel volume is accompanied by a decrease in cell residence volume in a scaffold [8]. Moreover, decreasing channel width increases the risk of thrombus formation when the network is surgically connected to the host vasculature and blood flows through the channel *in vivo*. Therefore the design of 3D vascular networks was determined from the aspect of compromise between mass transfer efficiency and volume loss/thrombogenic potential. In this study, the thinnest channel diameter was designed as 790 μm (fabricated result: 570 μm), but the previous study on the development of indirect-SL technology [23] suggests that channels with diameters of several tens of microns (i.e., the size of an arteriole) could be easily fabricated with this technology. And the data regarding GPC and mechanical properties of PLGA scaffolds fabricated by the indirect SL technology showed no significant changes in biomaterial properties [23]. Considering PCL degrade more slowly due to the presence of five hydrophobic–CH2 moieties in its repeating unit, we expect that the fabrication procedures do not significantly affect the mechanical properties of PCL as well.

The significant increase of apoptotic cell death in *R*_*scaffold*_ represents that the cell death was induced in response to hypoxia. However, although the percentage of hypoxic cells was 78.2% in *R*_*scaffold*_, the percentage of apoptotic cell death was only 41.2%. Severe and prolonged hypoxia may initiate apoptosis, whereas cells often adapt to acute and mild hypoxia and survive [35]. In this study, the positive hypoxic staining indicated that cells were alive under hypoxic conditions (oxygen concentration < 14 μM) but it could reveal neither the severity nor duration of hypoxia.

The patency of *VN*_*collagen layer*_ was maintained distinctly longer than *VN*_*non-collagen layer*_, but occlusion occurred after 24 h of whole-blood circulation in *VN*_*collagen layer*_. This difference was presumably caused by the absence of endothelium on the lumen and perturbed hemodynamics in *VN*_*non-collagen layer*_. The thin collagen layer plays a role as a barrier on the lumen and helps endothelial cells to reside on the lumen rather than inside the porous scaffolds (Fig 5c and 5d). This endothelium makes the lumen surface to be anti-thrombogenic. However, PCL exposed on the lumen of *VN*_*non-collagen layer*_ is not considered to contribute to the rapid thrombosis formation because it is a well-known bio-inert polymer [36]. Instead, the porous and rough surface perturbed hemodynamics such as shear, collision of blood element with the wall, and prolonged contact, which resulted in thrombus formation [37,38]. And the bubbles formed in *VN*_*non-collagen layer*_ generated extremely high air-blood interfaces and aggravated thrombosis [39]. However, the results from the whole-blood perfusion indicate that the artificial vascular networks cannot function as long-term mass transport channels *in vivo*. The artificial vascular networks must be combined with induction of microvessel formation by biomolecular cues and vascular cells to reduce the time required for regeneration of large-scale multicellular organs, thereby reducing the risk of thrombus formation.

This study focused on maximizing cell viability rather than specific functions by introducing fluidic channels in scaffolds. Fibroblasts are suitable for this purpose due to the ease of handling, abundant data about the metabolic properties which have been identified by researchers [31,40]. However, further study is required to investigate the effect of oxygen transport on cellular functions and potential for regenerating specific organs such as liver, lung and kidney.

## Conclusion

This study demonstrated that the modification of indirect-SL technology made it possible to construct artificial vascular networks in a porous scaffold by introducing hydrogel barrier on the luminal surface. This fabrication technology has the advantages of indirect-SL such as construction of 3D freeforms, wide selectivity of biomaterials, and compatibility with various other methods. The artificial vascular networks functioned as channels for oxygen transport, reducing the hypoxic volume and preventing apoptotic cell death. Furthermore, the endothelium of the vascular networks significantly retarded the occlusion of channels during whole-blood circulation. As the technology matures, the tissue mimics can be used as *in vitro* platforms to examine the physiologic and pathologic phenomena through vascular architecture. Furthermore, incorporation of microvessel formation in response to biomolecular cues and vascular cells, may allow large-scale formation of multicellular organs and reduce the risk of thrombus formation.
